# Effects of Cerium Doping on the Mechanical Properties and Energy-Releasing Behavior of High-Entropy Alloys

**DOI:** 10.3390/ma15207332

**Published:** 2022-10-20

**Authors:** Yusong Ma, Liang Zhou, Kaichuang Zhang, Xiqiang Gai, Jinyan He, Xinggao Zhang

**Affiliations:** State Key Laboratory of NBC Protection for Civilian, Beijing 102205, China

**Keywords:** high-entropy alloys, cerium doping, mechanical behavior, energy releasing

## Abstract

Energetic structural materials play an important role in improving the damage performance of future weapons. To improve the energy-releasing behavior, Al_0.5_NbZrTi_1.5_Ta_0.8_Ce_x_ high-entropy alloys were prepared by vacuum-arc melting. The results showed the presence of BCC and FCC phases in the alloy with dendritic-morphology-element segregation and there were significant dislocations in the alloys. The current study focused on the effects of cerium content on the dynamic compressive mechanical and energetic characteristics. Cerium doping enhanced the energy-releasing characteristics of high-entropy alloys. The severity of the reaction increased with the increase in the cerium content, while the dynamic compressive strength generally decreased with the increase in cerium content. The Al_0.5_NbZrTi_1.5_Ta_0.8_Ce_0.25_ showed excellent mechanical and energy-releasing characteristics. The ballistic experiments indicated that Al_0.5_NbZrTi_1.5_Ta_0.8_Ce_0.25_ can penetrate 6-millimeter A_3_ plates and ignite the cotton behind the target at a velocity of 729 m/s, making it an ideal energetic structural material.

## 1. Introduction

Traditional inert fragments mainly destroy targets through mechanical penetration, with limited effectiveness. The emergence of reactive fragments [1] has broken this trend. Current reactive fragmentation materials can be classified as metal–polymer [2,3], metal–metal oxide [4], metal–metal [5,6], etc. They do not meet the requirements of limited strength or energy-releasing characteristics. High-entropy alloys provide a solution to this problem through the flexibility of their composition design.

High-entropy alloys are characterized by high mixing entropy, severe lattice distortion and sluggish diffusion effect, which distinguish them from traditional metals with excellent properties such as high strength [7], high hardness [8], corrosion resistance [9], etc. High-entropy alloys have attracted the attention of scholars and some studies have been conducted on high-entropy alloys in the field of energetic structural materials. Zhang [10,11] investigated the microstructure and mechanical properties of a series of HfZrTiTa_x_ high-entropy alloys by modulating the Ta elements to achieve different combinations of strength and plasticity, pointing out their potential applications as energetic structural materials. Wang [12] introduced the transformation-introduced plasticity (TRIP) and transformation-introduced strength effect into NbZrTiTa high-entropy alloys to achieve high strength and certain plasticity at the same time. Dai’s team analyzed the energy-release process and overpressure of two types of high-entropy alloy fragments, WFeNiMo [13] and FeNiCoCr [14], in the velocity range, of 500–1800 m/s. It was shown that compared with tungsten alloy, WFeNiMo high-entropy alloy has better energy-releasing characteristics. Ren [15] analyzed the mechanism of compression and energy release of TiZrNbV alloy by numerical simulation and experiments. However, the energy-release threshold of the existing high-entropy-alloy energetic-structure materials is relatively high, regardless of whether it is measured by strain rate or impact velocity. Furthermore, chemically active rare-earth elements have not been doped into high-entropy alloys as energetic structural materials.

As a rare-earth element, cerium is chemically active. To balance the trade-off between mechanical properties and energetic characteristics, we introduced cerium into the Al_0.5_NbZrTi_1.5_Ta_0.8_ matrix and investigated the effect of cerium content on the dynamic compressive mechanical properties and energy-releasing characteristics of Al_0.5_NbZrTi_1.5_Ta_0.8_Ce_x_ high-entropy alloy.

## 2. Materials and Methods

### 2.1. Preparation of Al_0.5_NbZrTi_1.5_Ta_0.8_Ce_x_ High-Entropy Alloy

Al, Nb, Zr, Ti, Ta shots had a particle size of 3–6 mm and purity over 99.95% (Beijing Yijin New Material Technology Co., Ltd., Beijing, China). Ce was a block with purity over 99.95% (Beijing Yijin New Material Technology Co., Ltd).

Al_0.5_NbZrTi_1.5_Ta_0.8_Ce_x_ (x = 0, 0.25, 0.53 and 1.6, representing cerium (*at.* %) = 0, 5, 10 and 25, abbreviation CeX) alloys were prepared by vacuum-arc furnace (WK-II A, Beijing Physcience Opto-electronics Co., Ltd., Beijing, China) from a mixture of raw materials in a Ti-gettered argon atmosphere to prevent the alloy from being oxidized during the melting process. To achieve a homogeneous distribution of the elements in the alloy, the ingots were flipped and remelted 12 times. The as-cast alloys resembled buttons and were cut into three different types of cylindrical specimen, measuring Φ3 × 6 mm, Φ5 × 4 mm and Φ10 × 11 mm, using a wire-cutting method.

### 2.2. Microstructure Characterization

Crystal structures of the Al_0.5_NbZrTi_1.5_Ta_0.8_Ce_x_ alloys were analyzed by X-ray diffractometry (XRD, Bruker D8 Focus, Bruker, Karlsruhe, Germany) using Cu Kα target with 2θ between 20–80° and a scan step of 0.05°. The microstructures were examined by scanning electron microscopy (SEM, JEOL JSM 7200F, JEOL Ltd., Tokyo, Japan) equipped with energy-dispersive detectors (EDS, Oxford X-Max, Oxford Instruments, Abingdon, Britain). Fine-structure observations were conducted by transmission electron microscopy (TEM, FEI Tecnai G2 F20, FEI Company, Hillsboro, OR, USA) equipped with energy-dispersive detectors (EDS, XFlash 6|100, Bruker, Karlsruhe, Germany).

### 2.3. Performance Test

The quasi-static compressive mechanical property at room temperature was tested using a universal testing machine (MTS E43.504, Mechanical Testing & Simulation, Eden Prairie, MN, USA) with a specimen size of Φ3 × 6 mm and an initial strain rate of 10^−3^ s^−1^.

The dynamic compressive mechanical properties at different strain rates were tested using a Split Hopkinson pressure bar (SHPB, ATL1500, Archimedes Industry Technology Co., LTD, Beijing, China) with the specimen dimension of Φ5 × 4 mm. A high-speed camera was used to record the progress of compression. Strain, stress and strain rate can be obtained from the following equations:

(1) $$\sigma =\frac{\mathrm{AE}}{A_{0}}\varepsilon_{t}$$

(2) $$\varepsilon =-\frac{{2c}_{0}}{l_{0}}\overset{t}{\underset{0}{\int }}\varepsilon_{t}d\tau$$

(3) $$\dot{\varepsilon }=-\frac{{2c}_{0}}{l_{0}}\varepsilon_{r}$$

where E is the modulus of elasticity of the rod; c_0_ is the wave velocity of the stress wave; A and A_0_ are the cross-sectional area of the rod and the specimen; l_0_ is the length of the specimen; and ε_t_ and ε_r_ are the strains of the incident rod and transmitted rod, respectively.

Ballistic-gun (Nanjing University of Science and Technology, Nanjing, China) tests were used to investigate the penetration behavior and energy-releasing characteristics of high-entropy alloy. The specimen dimensions were Φ10 × 11 mm. During the test, the velocity of fragments was controlled in the range of 600–1000 m/s by changing the amount of propellant. The target was 6-millimeter A_3_ plate with cotton placed behind it. At the same time, a high-speed camera (Phantom, Vision Resesarch, Inc., Wayne, NJ, USA) was used to record the penetration and damage process of the fragments.

## 3. Results and Discussion

### 3.1. Microstructures

**Figure 1:**
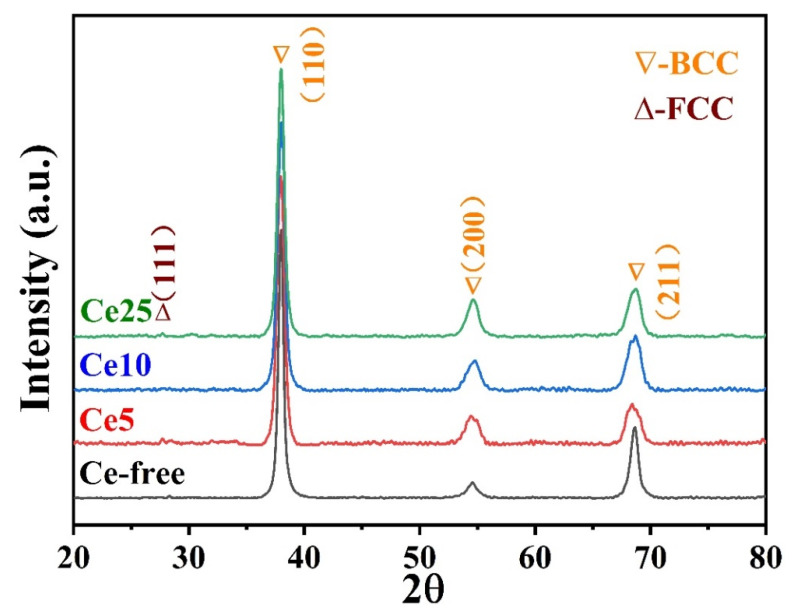
XRD patterns of CeX.

As shown in Figure 1, only one set of diffraction peaks corresponding to the body-centered cubic (BCC) structure was observed in the Ce-free alloy. However, when cerium was introduced into the matrix, a diffraction peak at 27.8° appeared and, compared with the Ce-free alloy, the diffraction peaks shifted towards high angles. The reason for this is that cerium has a large atomic size (181.8 pm) and, thus, reduces the lattice constant. Simultaneously, this indicated that cerium was introduced into the matrix successfully. By analyzing the SAED patterns, we determined that region IV had a BCC structure and region V had a face-centered cubic (FCC) structure. The peak at 27.8° corresponded to the FCC structure.

**Figure 2:**
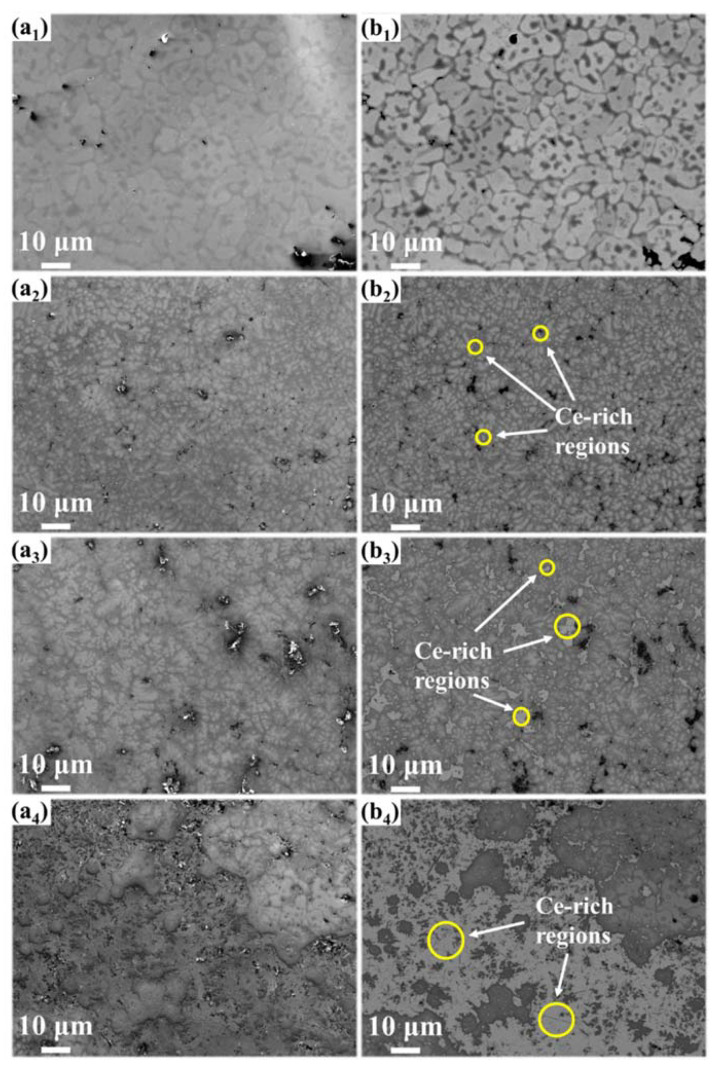
SEM (**left**)-BSE (**right**) images of CeX: (**a_1_**,**b_1_**) Ce-free; (**a_2_**,**b_2_**) Ce5; (**a_3_**,**b_3_**) Ce10; (**a_4_**,**b_4_**) Ce25.

**Figure 3:**
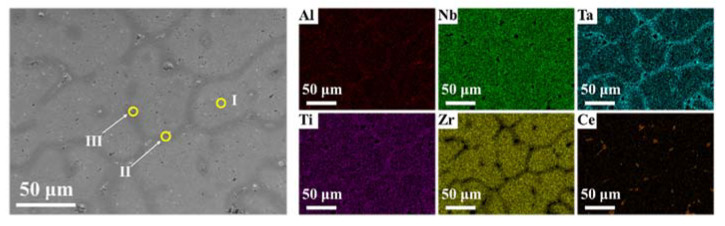
SEM and corresponding EDS images of Ce5: Region I: intra-dendrite region; Region II: inter-dendritic region and Region III: Ce-rich region.

The SEM-BSE image (Figure 2) shows that the CeX had a dendritic morphology. The addition of cerium made the dendrite fine. As the atom content of the cerium increased from 5% to 10%, the Ce-rich regions became larger and the number of holes on the surface increased. As the atom content of the cerium increased from 10% to 25%, the Ce-rich regions became larger and formed a continuous phase of cerium. The difference in contrast indicates the presence of three different regions. According to the EDS results (Figure 3), there was clearly elemental segregation in the alloys. The elemental distribution of each region is listed in Table 1. The intra-dendrite region (Figure 3, region I) and the inter-dendrite region (Figure 3, region II) differ in their elemental content. In the inter-dendritic region, the low-melting-point elements were prone to segregation, with Al, Ti and Zr having lower melting points than Ta and Nb; therefore, their content was higher than in the intra-dendritic region. Ce mainly segregated in the inter-dendrite region, forming region III (Figure 3, region III).

According to the research by Fu and Jin [16,17], the addition of cerium could encourage the formation of element segregation. In addition, for systems before and after mixing, the change in Gibbs free energy plays an important part [18]. During the cooling process, the temperature decreases and the Gibbs free energy of the system increases, resulting in spontaneous elemental segregation.

**Figure 4:**
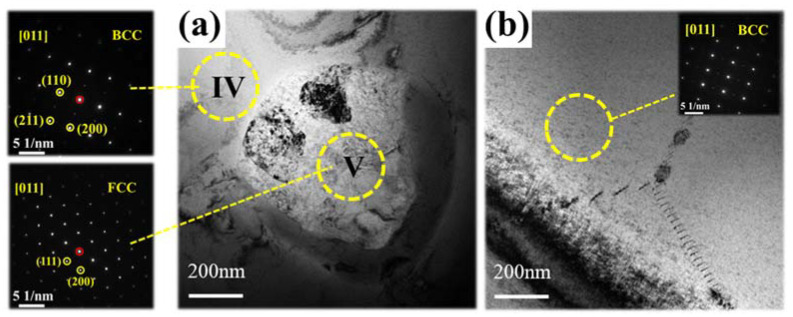
TEM images of Ce5: (**a**) Different regions and corresponding SAED patterns: Region IV: matrix and Region V: Ce-rich region and (**b**) dislocations in the alloy.

TEM images (Figure 4a) show the presence of two different regions (region IV and region V) with different structures in the alloy. According to the EDS results (Table 2), region IV, with a BCC structure, was rich in Al, Nb, Zr, Ti and Ta but less in Ce. Region V, with a FCC phase, was rich in Ce but contained small amounts of other elements. The Ce-rich areas were mainly present in the form of dots (approximately 50 nm), with few large Ce-rich areas. Based on the SEM image (Figure 3), it is assumed [17] that the dotted Ce-rich regions were mainly present in intra-dendrite region, while the large Ce-rich regions were present in inter-dendrite region. This result was observed because cerium has a low solid solubility in the matrix and tends to form another phase, corresponding to previous studies [19,20]. In addition, there were plenty of dislocations in the alloy (Figure 4b).

### 3.2. Mechanical Properties

**Figure 5:**
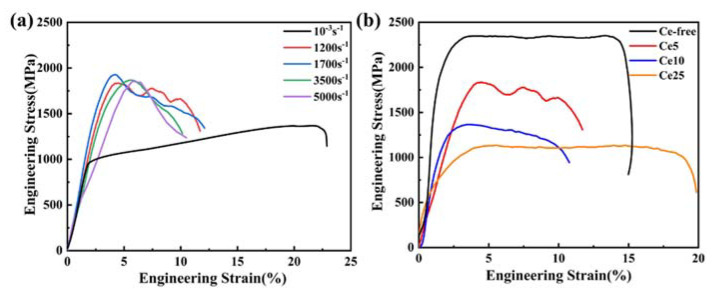
Mechanical behavior of CeX: (**a**) Compressive-engineering-stress-versus-engineering-strain curves of Ce5 at different strain rates and (**b**) compressive-engineering-stress-versus-engineering-strain curves of CeX at 1200 s^−1^.

The compressive-engineering-stress-versus-engineering-strain curves of the Ce5 at strain rates ranging from 10^−3^ s^−1^ to 5000 s^−1^ are shown in Figure 5a. Under quasi-static compression, the yield strength, compressive ultimate strength and fracture strain were 1000 MPa, 1367 MPa and 23%, respectively. At the beginning of loading, the alloy showed linear elastic characteristics. With the increase in strain, the resistance to deformation continued to increase and the material underwent work hardening. In this stage, internal micropores and microcracks accumulated along the grain boundaries. After reaching the highest compressive strength, the stress began to fall until the specimen fractured, at which point it was essentially the growth and aggregation of defects within the material. Under dynamic compression, the yield stage was not obvious. Instead, it was slowly unloaded in the form of strain softening after the stress reached the highest compressive strength, indicating a reduction in material plasticity. This was due to the lattice distortions present in the high-entropy alloy because of the inevitable differences between the elements. The strong internal stresses caused the brittleness of the alloy. Furthermore, under quasi-static conditions with a low strain rate, there was sufficient time for the dislocations to slip and cluster. Increasing the strain rate reduces the interaction time between dislocations and suppresses the movement of the dislocation slip and clustering, resulting [21] in brittle alloys.

The stress–strain curves of these three kinds of alloys at the strain rate of 1200 s^−1^ are shown in Figure 5b. When the atomic percentage of cerium increases from 5% to 25%, the yield strength and ultimate compressive strength decrease. According to the law of mixing, the addition of soft cerium reduces the strength of alloys. However, on the other hand, the segregation of cerium atoms at dislocations could improve the resistance of dislocation movement, inhibit migration [16] and enhance yield strength. In addition, cerium can cause large lattice distortion [22] because of the large atomic radius, which can induce solid-solution strengthening. This phenomenon is caused by the trade-off between these factors. The fracture strain, on the other hand, differs considerably, with the fracture strain of Ce25 reaching 19%, much higher than the other two materials.

**Figure 6:**
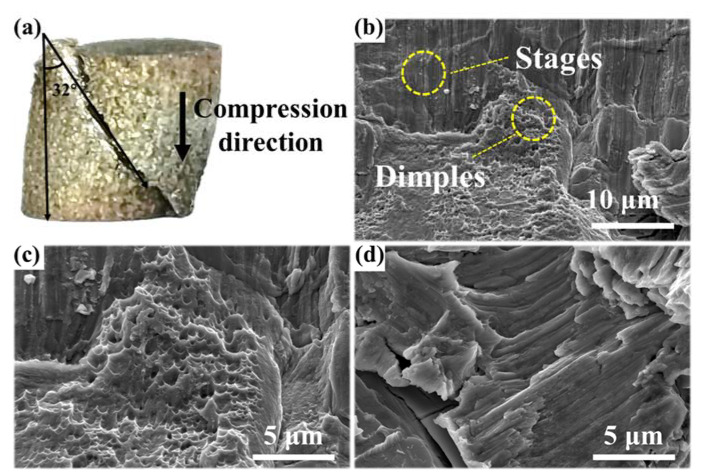
Fractured specimen after quasi-static compression. (**a**) Macro-feature image. (**b**) SEM images of micro-fracture surface of Ce5. (**c**) Dimples and (**d**) stages under quasi-static compression.

Figure 6 shows the macro and micro features of the Ce5 under quasi-static compression. The alloy exhibited the characteristics of brittle fracture macroscopically. The compression direction was at an angle of 32° to the crack (Figure 6a). Figure 6b–d shows the SEM images of the micro-fracture surface. The dimples were distributed on the fracture surface. Generally, dimples are characteristic of ductile fracture and the stages result from brittle fracture; this indicates the presence of a ductile and brittle fracture mechanism in the Ce5.

### 3.3. Energetic Characteristics

#### 3.3.1. Dynamic Compression Process

**Figure 7:**
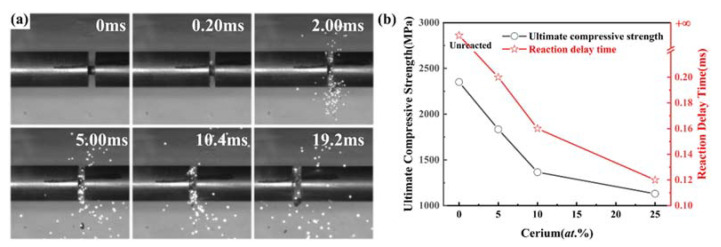
(**a**) Dynamic compression process of Ce5 at 1200 s^−1^ and (**b**) ultimate compressive strength and reaction delay time of CeX.

The compressive progress of the Ce5 specimens at 1200 s^−1^ was recorded by a high-speed camera to assess their energetic characteristics. As shown in Figure 7a, when the specimens were compressed, they broke up and reacted violently with the air. This can be explained by the “hot spot” theory [23]. When the specimen was subjected to impact, the alloy was compressed and deformation occurred. The internal accumulation of deformation energy and frictional heat led to a sharp temperature increase, providing energy for “hot spots”. As the reaction proceeded, the fragment dispersed in all directions and the violence of the reaction peaked. Subsequently, the flame gradually faded until it disappeared.

We defined t_0_ as the moment when the high-entropy alloy specimen started to become compressed and the reaction delay time as the time interval between when the specimen started to become compressed and when the reaction started. As shown in Figure 7b, the reaction delay times for the CeX ranged from 0.12 ms to 0.2 ms. As the cerium content increased, the reaction delay time shortened and the reaction reached its most violent moment earlier, indicating that the doping of the cerium made the energy-release reaction easier to produce. Furthermore, the energy-release reaction became more violent with the cerium-content increase.

#### 3.3.2. Ballistic Performance

**Figure 8:**
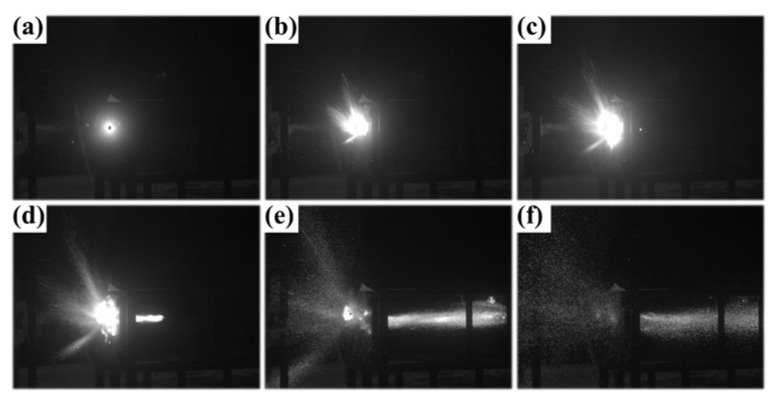
Ce5 fragment penetrating a 6-millimeter A_3_ plate: (**a**) 0.1 ms, (**b**) 0.4 ms, (**c**) 0.7 ms, (**d**) 1.3 ms, (**e**) 4.1 ms and (**f**) 9.8 ms.

Figure 8 shows the process of the Ce5 fragment penetrating the 6-millimter A_3_ plate at the speed of 729 m/s. Some cotton was placed behind the target to evaluate the post-target damage effect. When the Ce5 fragment impacted the target, the mechanical impact triggered a violent energy-release reaction from the material. Flame was generated at the striking position of the fragment on the plate and formed a huge conical expansion region. The fragment broke into small pieces and spread out in all directions. Some of the burning pieces penetrated the target and the cotton behind the target was ignited.

**Figure 9:**
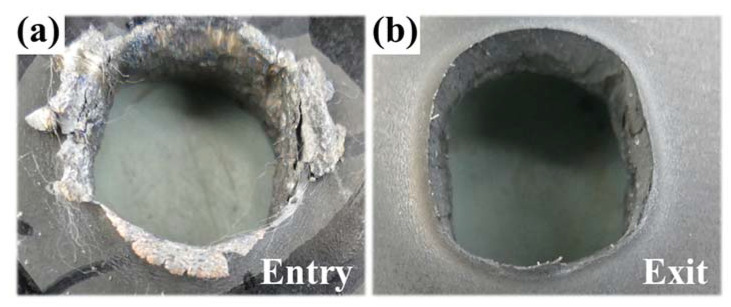
Holes formed in the process of the fragment penetrating A_3_ plate: (**a**) Entry and (**b**) Exit.

By analyzing the holes formed in the process of the fragment penetrating the target, as shown in Figure 9, it can be observed that the diameter of the hole was larger than that of the fragment. Furthermore, traces of melting can be observed, indicating the violent reaction of the fragment and the temperature exceeding the melting point of the steel plate. On the back of the target, a rolled edge occurred around the hole. It is supposed to have been caused by the overpressure generated by the deflagration reaction of the fragment, which makes the surrounding air expand rapidly and, thus, tear the edge of the hole.

## 4. Conclusions

In conclusion, Al_0.5_NbZrTi_1.5_Ta_0.8_Ce_x_ the high-entropy alloys prepared by vacuum-arc melting showed excellent compressive mechanical properties and energetic characteristics. The following conclusions can be drawn:

(1) The XRD patterns indicate that the matrix had a BCC structure. Cerium doping resulted in the appearance of FCC structure in the alloys. The SEM and EDS illustrated dendritic morphology and element segregation was present in the alloy. The TEM images showed the presence of regions rich in Al, Nb, Zr, Ti and Ta with BCC structures and regions rich in Ce with FCC-structure regions and dislocations in the alloy.
(2) The cerium-atom percentage has a significant influence on mechanical properties and energy-releasing behavior. As the cerium-atom percentage increased from 5 *at.* % to 25 *at.* %, the dynamic compressive yield strength and ultimate compressive strength showed a generally decreasing trend. The fracture strain of the Ce5 and Ce10 ranged from 9% to 10%, increasing to 19% of the Ce25 because of the continuous Ce-rich regions with FCC structures. According to the images from the high-speed camera, cerium doping improved the energy-releasing characteristics. As the content of cerium increased, the reaction-delay time became shorter. The time to reach the most violent reaction shortened and the energy-release reaction became more violent.
(3) Al_0.5_NbZrTi_1.5_Ta_0.8_Ce_0.25_ can penetrate 6-millimeter A_3_ plates at a speed of 729 m/s and ignite cotton behind a target. It is an ideal energetic structural material because of its combination of excellent mechanical properties and energetic characteristics.

## Figures and Tables

**Table 1:** Element distribution in different regions in SEM images.

Element	Region I (*at.* %)	Region s (*at.* %)	Region III (*at.* %)
Al	4.5	9.0	7.6
Nb	28.3	21.9	4.1
Zr	11.3	21.5	3.1
Ti	27.0	31.4	5.4
Ta	28.5	16.0	6.0
Ce	0.4	0.3	73.9

**Table 2:** Element distribution in different regions in TEM images.

Element	Region Ⅳ (*at.* %)	Region Ⅴ (*at.* %)
Al	4.9	0.0
Nb	23.4	2.5
Zr	25.4	2.4
Ti	29.8	2.9
Ta	15.7	1.1
Ce	0.8	91.1

## Data Availability

The data presented in this study are available on request from the corresponding author.
